# Prevalence of psychosocial distress in school going adolescents in rural Pakistan: findings from a cross-sectional epidemiological survey

**DOI:** 10.1192/bjo.2021.196

**Published:** 2021-06-18

**Authors:** Syed Usman Hamdani, Zill-e- Huma, Hashim Javed, Azza Warraitch, Atif Rahman, Asad Tameezuddin Nizami, Fareed Aslam Minhas

**Affiliations:** 1 Institute of Psychiatry, Benazir Bhutto Hospital, Department of Primary Care and Mental Health, University of Liverpool, Global Institute of Human Development, Shifa Tameer-e-Millat University, Human Development Research Foundation; 2 Department of Primary Care, University of Liverpool, Global Institute of Human Development, Shifa Tameer-e-Millat University, Human Development Research Foundation; 3 Global Institute of Human Development, Shifa Tameer-e-Millat, University, Human Development Research Foundation; 4 Human Development Research Foundation; 5 Department of Primary Care and Mental Health, University of Liverpool; 6 Institute of Psychiatry, Benazir Bhutto Hospital; 7 Global Institute of Human Development, Shifa Tameer-e-Millat University

## Abstract

**Aims:**

Early interventions are recommended in adolescents to prevent long-term psychiatric morbidity. However, in Low and Middle Income Countries (LMICs), where there are no child and adolescent mental health services, early identification of adolescents at-risk of mental health problems remains a challenge. Pediatric Symptoms Checklist (PSC) is used in preventive child healthcare services in a number of high income countries for early identification of children and adolescents in need of mental health services. The aim of this study was to assess the reliability and validity of self-rated, Urdu version of PSC to identify at-risk adolescents studying in the public schools of rural Rawalpindi in Pakistan.

**Method:**

We did a cross-sectional epidemiological survey with all adolescents aged 13–15 years, studying in 41 public schools of Kallar Syedan sub-district in Rawalpindi, Pakistan. An adapted Urdu version of self-reported PSC was used to assess the psychosocial distress in adolescents in-terms of externalizing, internalizing and attention problems. Strengths and Difficulties Questionnaire (SDQ) was used as a gold standard measure. Youth version of PSC and SDQ were administered in classroom settings by trained research teams.

**Result:**

The data were collected from 5856 adolescents (response rate 97%) between April-May, 2019. The mean age of the participants was 14.37 years (±1.06); 51% participants were female. The internal consistency reliability of Urdu version of PSC was good (Cronbach alpha 0.85). At the standard cut-off score of PSC ≥28, the prevalence rate of psychosocial distress in adolescents was 25.5% (27.4% in boys & 23.6% in girls). Using the SDQ total difficulties score ≥16 as a standard criterion; the area under the ROC curve was 0.85 (95% CI 0.82–0.88), with a sensitivity of 57.64% and specificity of 89.10% of PSC. If the sensitivity and specificity of PSC is optimized to 76% at the cut-off score of PSC ≥ 24, the prevalence rates of psychosocial distress in adolescents is increased to 41%.

**Conclusion:**

In our study, 1 in 4 adolescents in public schools of rural Rawalpindi in Pakistan have been identified at-risk of poor socio-emotional development. Urdu version of PSC is a reliable and valid tool to identify adolescents in need of psychosocial interventions in public schools of rural Pakistan. While the standard cut-off score yields a better specificity; PSC with relatively lower cutoff score can be used a screening tool to identify at-risk adolescents in public schools of rural Pakistan.

